# The Value of Texas’ Hot Waters

**Published:** 1899-05

**Authors:** H. C. Black

**Affiliations:** Waco, Texas


For Texas Medical Journal.

### The Value of Texas' Hot Waters.

BY H. C. BLACK, M. D., WACO, TEXAS.

The value of water as a therapeutic agent is being yearly better recognized. Time was, a generation since, that a patient, racked and burning with fever, was oft times denied a drink of cold water as though it were poison. Time has changed our views; today the fevered sufferer is given cold water to drink, and is sponged, wet packed, or bathed in water, either warm, hot, or cold, as the exigencies require. The fever is cooled, the patient made comfortable, and the stomach not filled with drugs, but in a condition to take food with hope of digesting it.

But it is not my purpose to call the attention of physicians to the value of hydrotherapy in the treatment of disease; that is an acknowledged fact.

We all have patients come to us for advice as to where they had best go to secure most benefits from the waters of various springs and wells in America, Mexico and Europe. It seems to be a time-honored custom to advise many people to try Hot Springs, Arkansas; Topo Chico Springs, at Monterey, Mexico; and dozens of other similar places. Why send a patient suffering with chronic rheumatism, or palludal poison, or syphilitic poison, or a dozen other diseases requiring the benefits of hot baths and skilled attendants, out of the State of Texas to be cured? Texas has the hot waters flowing from the ground in abundance at exactly the right temperature for such baths, 99° to 103°. I made a visit to Hot Springs, Arkansas, to discover, if I could, any superiority of the waters there over the hot waters in the wells at Waco, Texas. I found the waters of Hot Springs pour out of the mountain at a temperature of 135° F.; so hot no man could bathe in it until it is cooled by the addition of cold water. The baths are taken in Hot Springs at a temperature ranging from 99° to 103°; now this is exactly the *natural* temperature of the waters in the different wells at Waco. The well at Waco Natatorium flows from the ground into the pool or into the bath tubs at 102° F. Marlin, Texas, has also a hot well, temperature same as Hot Springs, Ark., 135° F.

I visited Topo Chico Hot Springs, at Monterey, Mexico, and my candid conclusion is, any disease that can be cured by aid of the hot water of either Topo Chico or Hot Springs, Ark., can also be cured just as quickly by the hot waters at either Waco or Marlin, Texas.

I have personal knowledge of a large number of people who have been cured by the hot baths of Waco of chronic rheumatic affections, mercurial and lead poisoning, skin diseases, and other troubles of a similar character. Why should we send persons so affected out of the State of Texas for treatment, when they can be cured at home and the hundreds of thousands of dollars they spend elsewhere be kept in the State.

There is not a *better* hotel in the city of Hot Springs than two or three of Waco's hotels. There is not a hotel in Monterey, Mexico, equal to half a dozen of Waco hotels. The hotel under the same roof as Waco Natatorium is not surpassed in Texas for excellence of table or elegance of appointment. I also understand Marlin will build again her hotel recently burned at her natatorium. Patients can be made as comfortable at either town as any place out of Texas. They can be cured as quickly; then why should Texas physicians send them out of the State?

